# Changes of serum IgG glycosylation patterns in rheumatoid arthritis

**DOI:** 10.1186/s12014-023-09395-z

**Published:** 2023-02-21

**Authors:** Xiaoyue Deng, Xiaomin Liu, Yan Zhang, Dan Ke, Rui Yan, Qian Wang, Xinping Tian, Mengtao Li, Xiaofeng Zeng, Chaojun Hu

**Affiliations:** 1 Department of Rheumatology and Clinical Immunology, Peking Union Medical College Hospital, Chinese Academy of Medical Sciences, Peking Union Medical College, Beijing, 100730 China; 2 National Clinical Research Center for Dermatologic and Immunologic Diseases (NCRC-DID), Ministry of Science & Technology, Beijing, 100730 China; 3 Key Laboratory of Rheumatology and Clinical Immunology, Ministry of Education, Beijing, 100730 China; 4 Medical Science Research Center (MRC), Peking Union Medical College Hospital, Peking Union Medical College and Chinese Academy of Medical Sciences, Beijing, 100730 China; 5 Department of Rheumatology, Shunyi District Hospital, Beijing, 101300 China

**Keywords:** RA, ILD, Immunoglobulin G, Lectin microarray, Glycosylation

## Abstract

**Background:**

RA is a common chronic and systemic autoimmune disease, and the diagnosis is based significantly on autoantibody detection. This study aims to investigate the glycosylation profile of serum IgG in RA patients using high-throughput lectin microarray technology.

**Method:**

Lectin microarray containing 56 lectins was applied to detect and analyze the expression profile of serum IgG glycosylation in 214 RA patients, 150 disease controls (DC), and 100 healthy controls (HC). Significant differential glycan profiles between the groups of RA and DC/HC as well as RA subgroups were explored and verified by lectin blot technique. The prediction models were created to evaluate the feasibility of those candidate biomarkers.

**Results:**

As a comprehensive analysis of lectin microarray and lectin blot, results showed that compare with HC or DC groups, serum IgG from RA patients had a higher affinity to the SBA lectin (recognizing glycan GalNAc). For RA subgroups, RA-seropositive group had higher affinities to the lectins of MNA-M (recognizing glycan mannose) and AAL (recognizing glycan fucose), and RA-ILD group had higher affinities to the lectins of ConA (recognizing glycan mannose) and MNA-M while a lower affinity to the PHA-E (recognizing glycan Galβ4GlcNAc) lectin. The predicted models indicated corresponding feasibility of those biomarkers.

**Conclusion:**

Lectin microarray is an effective and reliable technique for analyzing multiple lectin–glycan interactions. RA, RA-seropositive, and RA-ILD patients exhibit distinct glycan profiles, respectively. Altered levels of glycosylation may be related to the pathogenesis of the disease, which could provide a direction for new biomarkers identification.

**Supplementary Information:**

The online version contains supplementary material available at 10.1186/s12014-023-09395-z.

## Background

Rheumatoid arthritis (RA) is one of the most prevalent systemic autoimmune diseases (AIDs), which has heterogenous symptoms featured by synovium hyperplasia, autoantibody production, such as RF (rheumatoid factor) and ACPA (anti–citrullinated protein antibody), as well as cartilage and joint destruction [1]. The etiology of RA is heterogeneous and influenced by environmental insults, susceptibility genes, epigenetic modifications, and post-translational modifications [2]. Extra-articular manifestations occur in the RA population and the most frequent one is interstitial lung disease (ILD), with 14.7% occurrence rate within 5 years, and RA-ILD worsens the disease prognosis and contributes to the excess morbidity and mortality of RA patients [3].

During the last decades, the diagnosis and management of RA patients have been mainly dependent on autoantibody measurements, especially RF and ACPA, and the presence of autoantibodies is associated with more severe symptoms, joint damage, and increased mortality [4, 5]. Routine examinations of ILD and the disease progression in RA patients include clinical symptoms, lung function and high-resolution computed tomography, ultrasound of the lung, diffusion capacity for carbon monoxide of the Lung, etc. However, considering the importance of early detection for the management of patients with RA, biomarkers for early diagnosis of RA-ILD are still challenging and require further investigation [3].

Protein glycosylation, one of the most common post-translational modifications, has profound effects on protein stability, activity and functions, which greatly results in the expansion of proteome [6]. The aberrant glycosylation indicates pro- or anti- inflammatory effects so that they have been considered as biomarkers for many autoimmune diseases [7–9]. Of note are the glycosylation abnormalities in immunoglobulin G (IgG), which is the most abundant glycoprotein in human serum [10, 11]. Numerous studies have confirmed the importance of glycosylation involved in the pathogenesis of RA. For example, deceased serum ACPA-IgG galactosylation, increased serum matrix metalloproteinase-3 α-2,6-sialylation, acute-phase proteins galactosylation and fucosylation were observed in the patients of RA [12–15]. Therefore, studies on glycoproteomic profile and alteration in terms of different physio-pathological states may support the discovery of candidate biomarkers for RA patients, especially for the diagnosis of ILD.

As carbohydrate binding proteins, lectins are categorized based on their specificity for monosaccharide units. The ability of these carbohydrate-binding proteins to distinguish different glycan structures have made them very useful for glycosylation analysis. Lectin microarray is a panel of lectins that arrayed on a glass slide. By recognizing various glycans, it could detect changes in glycosylation pattern. As a high-throughput, high-speed, and high-specific platform for glycan analysis, it has been widely used for cancers and AIDs [16]. In the present study, lectin microarray technology containing 56 lectins was applied to decipher the serum IgG glycosylation profile in patients with RA. By validation with lectin blot and creation of predicted models, we provided several specific glycosylation alterations as candidate biomarkers for the diagnosis of RA as well as RA-ILD, indicating the underlying mechanisms of pathogenesis that related to glycosylation.

## Methods

### Patient and public involvement

Patients were involved in this study by donating blood at Peking Union Medical College Hospital from 2019 to 2021, when attending population surveys.

### Study cohort

A total of 464 serum samples collected from 214 RA patients; 150 disease controls (including 50 anti-phospholipid syndrome (APS) patients, 50 Takayasu arteritis (TA) patients and 50 vascular disease (VD) patients) and 100 healthy controls (HCs) were used for lectin microarray and lectin blot analysis. Patients with RA were divided into four subgroups according to their sera positivity for autoantibodies, complication of ILD, and disease activity score (DAS). Two ways of DAS standards (DAS28-ESR = 0.56*sqrt(TJC) + 0.28*sqrt(SJC) + 0.70*Ln(ESR) + 0.014*GH, DAS28-CRP = 0.56*sqrt(TJC) + 0.28*sqrt(SJC) + 0.36*Ln(CRP + 1) + 0.014*GH + 0.96) have been applied to categorize disease activity states of RA patients as high (DAS28 > 5.1), moderate (3.2 < DAS28 ≤ 5.1), low (2.6 < DAS28 ≤ 3.2) disease activity and remission (DAS28 ≤ 2.6). APS, TA, and VD patients constituted the disease controls (DCs). In the lectin blot verification analysis, a new cohort of samples was collected from 50 RA-ILD patients. All patients with RA were diagnosed based on the 1987 revised American College of Rheumatology criteria, and all patients with APS, TA and VD were diagnosed according to respective general criteria used for each disease [17]. Patients with other autoimmune diseases, cancers, infections, or any severe comorbidities were excluded. Information such as demographic data, initial symptoms, disease duration, history of allergies and physical examination was recorded. In the clinical context, ACPA autoantibodies are measured by the anti-cyclic citrullinated peptide (anti-CCP assay [18], and we defined seropositive as both RF and CCP positive while seronegative as the opposite). Serum samples were obtained by separation from peripheral blood and stored at − 80 °C until use. No sample was exposed to more than one freeze-thaw cycle before analysis. This study was approved by the Medical Ethics Committee of PUMCH (Beijing, China). All participants provided written informed consent.

### Lectin microarray

**Fig. 1:**
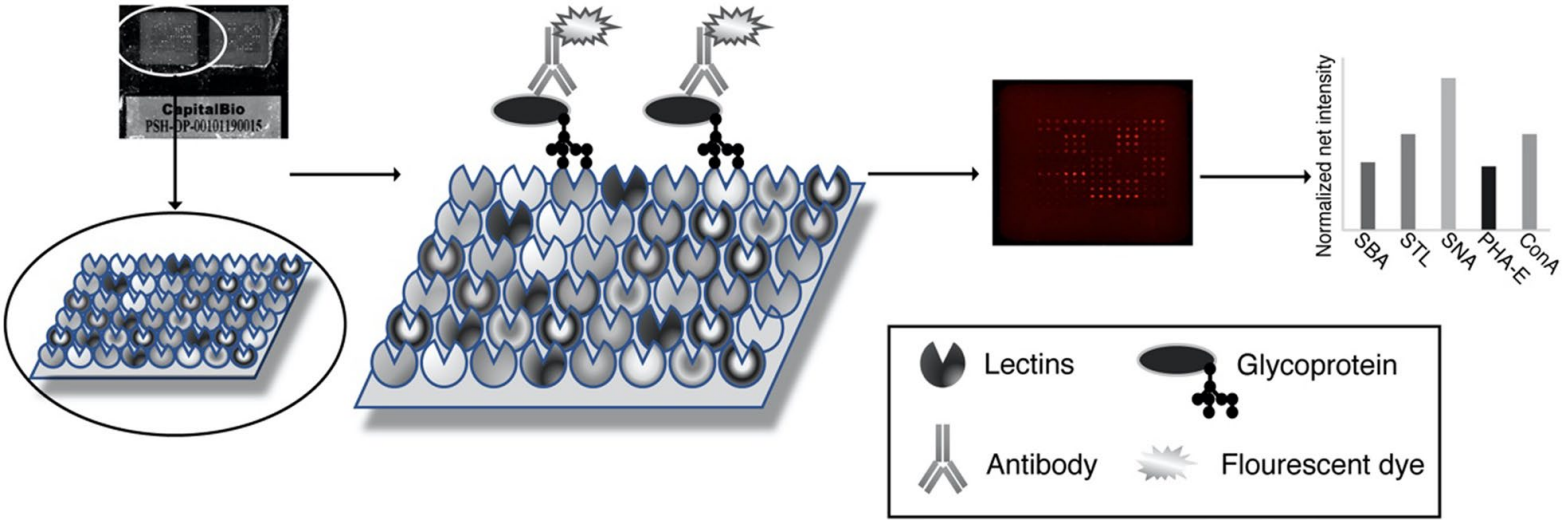
The methodology for the identification of lectin–glycan interaction was illustrated from left to right. Immobilized on a glass surface, lectins can be probed with unlabeled glycoprotein, which could be detected by fluorescently labeled antibody. The signals obtained from the lectin microarrays are subjected to statistical analysis. Based on the known location of the 56 lectins on the microarray (Fig. 1), identification of the interacting glycan can be done effectively

A commercial lectin microarray (BCBIO Biotech, Guangzhou, China) with 56 lectins, which can quickly and sensitively detect common glycan variants in IgG, was used to detect the glycopattern of serum IgG according to the previous protocol (Fig. 1; Additional file 1: Fig. S1) [19, 20]. Briefly, lectin microarrays were removed from − 80 °C and warmed up at room temperature for half an hour, then incubated with a blocking buffer (3% BSA in PBS) for 2 h accordingly. Microarrays were then washed with PBS three times and dried by spinning at 500 g for 5 min. Subsequently, 200 μl of the 1:1,000 diluted sera samples were applied to the microarray and incubated at 4 °C overnight. After washing three times with PBS, the microarrays were incubated with 5 ml Cy3-labeled goat anti-human IgG antibody (1:1,000 diluted; Jackson Immuno Research Labs, Pennsylvania, PA, USA) avoid light at room temperature for 50 min. Finally, after three times washing with PBS and twice washing with D.I. water, the microarrays were dried by spinning at 500 g for 5 min and scanned using the GenePix 4000B (Molecular Devices, Sunnyvale, CA) Microarray Scanner at a wavelength of 635 nm and a photomultiplier tube setting of 600.

### Lectin microarray data analysis

For lectin array assays, the GenePix Pro 6.0 software (Molecular Devices, Sunnyvale, CA) and proprietary gal files were used to extract the median foreground and background intensity values for each spot on the arrays. The signal-to-noise ratio (S/N) (the medium intensity of the spot foreground relative to the background) of each lectin spot was calculated. To reduce the bias of the lectin microarray in the inter-array, we normalized the S/N data in terms of quality control values between arrays [21]. In addition, we determined that there were significant differences in lectin binding between the test groups by using the method of Hu et al. [19, 20], and for the difference, lectin must meet the following two conditions: (a) fold change [group1 (S/N)/group2 (S/N)] ≥ 1.3 or < 0.77, (b) p value < 0.05.

### Lectin blot analysis

To validate the results of the lectin microarray, serum samples from different groups were randomly chosen from the lectin microarray analysis cohort and a new cohort of 50 RA-ILD patients was included. Briefly, to determine the location of IgG in immunoblotting, 1:100 diluted serum proteins mixed with loading buffer (CW biotech, Beijing, China) were separated by 10% sodium dodecyl sulphate–polyacrylamide gel electrophoresis (SDS-PAGE). The separated proteins were electro-transferred onto polyvinylidene fluoride membranes (Millipore, Billerica, MA, USA). After blocking non-specific binding sites with 10 × Carbo-Free Blocking Solution (1:10; Vector Laboratories Inc., US) at room temperature for 2 h, the membranes were incubated with 20 μg/mL Cy3 (1:1,000; GE Healthcare, Chicago, IL, USA)-labeled lectins including SBA, STL, PHA-E, SNA, Jacalin, SNA-I, MNA-M, AAL, ConA, PHA-L, DBA (EY Laboratories, Inc., US and Vector Laboratories Inc. US) at 4 °C overnight in the dark. Excess lectins were removed by washing three times with PBST. The washed and dried membranes were detected by a fluorescence signal system of Typhoon FLA 9500 (GE Healthcare, Chicago, IL, USA). Finally, ImageJ software was used for signal intensity analysis.

### Statistical analysis

SPSS 22.0 was used to perform statistical analyses, and GraphPad Prism 9 was used to drew plots in the study. Continuous variables were expressed as mean ± standard deviation. The differences among the RA, DC, HC groups were tested by one-way analysis of variance (ANOVA); the Student t-test was used to compare the subgroups in RA patients. The predicted models were evaluated by using the receiver operating characteristic (ROC) curve, which is obtained by calculating the sensitivity and specificity of the test at every possible cutoff point and plotting sensitivity (the proportion of true positive results) against 1-specificity (the proportion of false positive results). The method of Youden index (J) was employed to identify optimal cutoff points based on sensitivity, specificity, and the ROC curve. J was defined as the maximum vertical distance between the ROC curve and the diagonal or chance line and was calculated as J = maximum (sensitivity + specificity – 1). P value less than 0.05 was considered statistically significant.

## Results

### Patient characteristics

Among RA patients, 21% (46/214) were male, and the median age was 55 years old. RF ( +), CCP ( +), and seropositive were presented in 84% (179/214), 81% (174/214), and 77% (164/214), respectively. 60 of RA patients (28%) were complicated with ILD. RA patients were characterized as 75 (35%) of remission, 29 (14%) of low disease activity, 61 (29%) of moderate disease activity, 48 (22%) of high disease activity according to DAS28-ESR. And based on DAS28-CRP, RA patients were divided into 102 (48%) of remission, 21 (10%) of low disease activity, 57 (27%) of moderate disease activity, 30 (14%) of high disease activity, respectively. Among DCs, 28% (14/50) were male and the median age was 44.5 in APS, 4% (8/50) were male with median age of 39.5 in TA, 7% (14/50) were male and the median age was 61 in VD. In the group of HC, 81% (81/100) were male and the median age was 65. For the three disease controls, which are chronic inflammatory diseases without RA antibody reactivity, as well as HC, those items related to RA are not applicable.

### Analysis of serum IgG glycosylation in patients with RA by lectin microarray

**Fig. 2:**
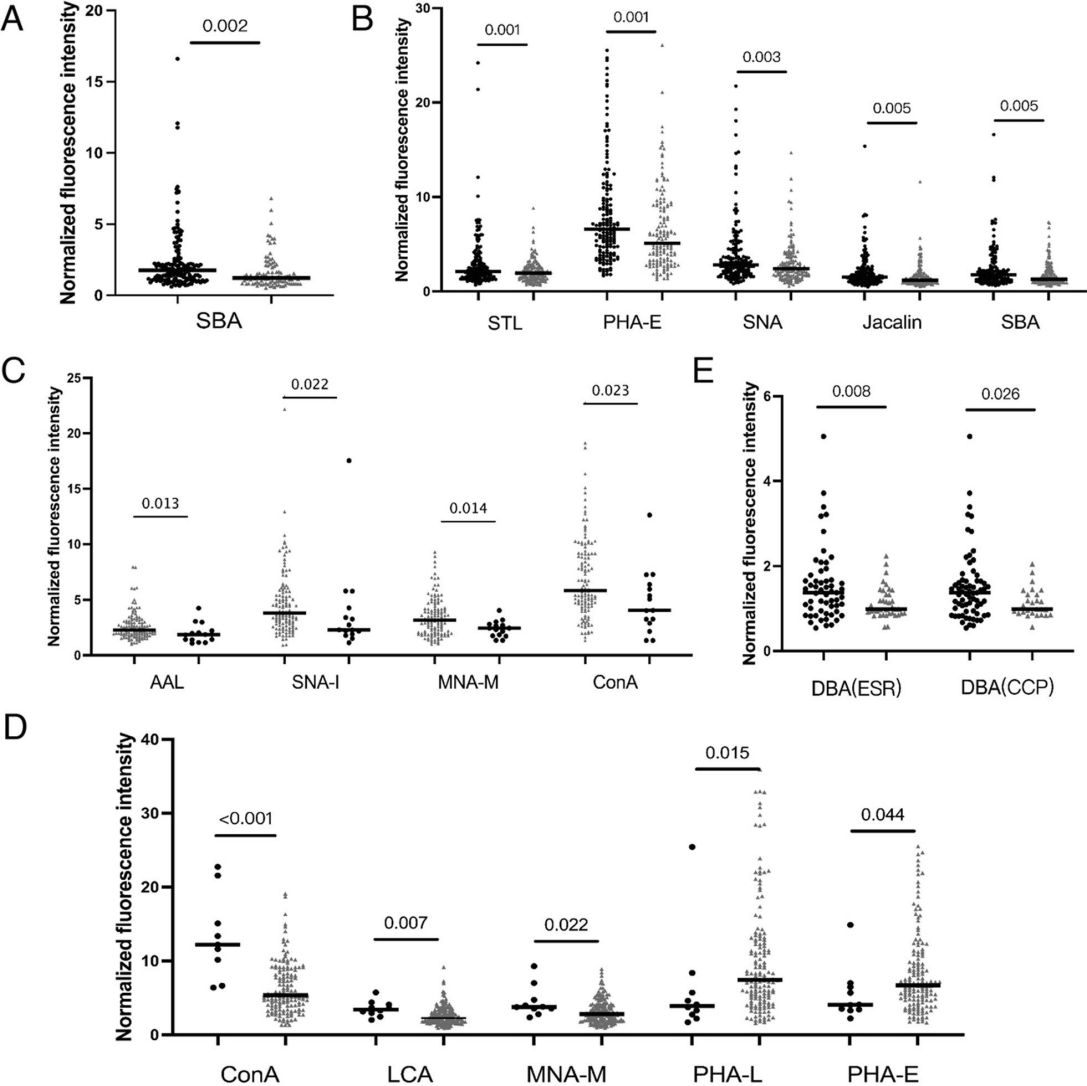
Specific changes of serum IgG glycosylation in groups. **A** left group in black refers to RA and right group in gray refers to HC; **B** each left group in black refers to RA and right group in gray refers to DC; **C** each left group in gray refers to seropositive and right group in black refers to seronegative; **D** each left group in black refers to RA-ILD and right group in gray refers to RA-nILD; **E** each left group in black refers to Remission and right group in gray refers to High Disease Activity. p values were showed on the top of each compared groups

Serum samples of 164 RA patients, 150 DC patients, and 100 HC were detected by lectin microarray (Fig. 1; Additional file 1: Fig. S1). Results of 56 lectins among all groups were performed cluster analysis and shown on a whole scope (Additional file 1: Fig. S2). In detail, 6 among 56 lectins showed significant differential signal intensities between the RA and DC/HC groups (Table 1). Serum IgG from RA patients had a higher affinity for SBA compared to HC, as well as higher affinities for STL, PHA-E, SNA, Jacalin, SBA compared to DC. Therefore, glycan levels of GalNAc (recognized by SBA), GlcNAc (recognized by STL), Galβ4GlcNAc (recognized by PHA-E), Sialic acid (recognized by SNA), Galβ3GalNAc (recognized by Jacalin) were increased characteristically in serum IgG from patients with RA (Fig. 2). Sugar specificity for lectins with significant differences between groups are listed in Table 2. Thus, the above lectins was chosen for verification in the later process.

**Table 1:** S/N data of lectin microarray

Group1/2	Name	p-value	Fc_Group1/2	Mean_1	Mean_2	Std_1	Std_2
RA/HC	SBA	0.002	1.44	2.34	1.62	2.14	1.16
RA/DC	STL	0.001	1.40	2.99	2.14	2.92	1.2
	PHA-E	0.001	1.30	8.36	6.41	6.05	4.24
	SNA	0.003	1.37	4.08	2.97	4.11	2.14
	Jacalin	0.005	1.32	2.06	1.57	1.78	1.25
	SBA	0.005	1.32	2.34	1.77	2.14	1.26
Seropositive/Seronegative	SNA-I	0.022	1.43	5.56	3.88	7.68	4.04
	MNA-M	0.014	1.45	3.46	2.39	1.74	0.73
	AAL	0.013	1.39	2.77	1.99	1.99	0.87
	ConA	0.023	1.50	7.25	4.82	4.68	2.91
ILD/nILD	ConA	0	2.49	15.64	6.29	9.14	3.42
	LCA	0.007	1.35	3.53	2.62	1.05	1.27
	MNA-M	0.022	1.42	4.49	3.16	2.11	1.6
	PHA-L	0.015	0.61	6.19	10.11	7.03	7.71
	PHA-E	0.044	0.64	5.46	8.55	3.64	6.13
R/HDA(ESR)	DBA	0.008	1.35	1.54	1.14	0.86	0.37
R/HDA(CCP)	DBA	0.026	1.32	1.50	1.14	0.80	0.34

Fc_Group1 vs Group2: fold change of S/N in Group1compare to Grouo2; Mean_Group: mean value of S/N; Std_Group: standard deviation; R: Remission HDA: High disease activity; S/N, the medium intensity of the spot foreground relative to the background

**Table 2:** Sugar specificity for lectins with significant differences between groups

Lectins	Groups with significant change	Lectin full name	Monosaccharide specificity
SBA	RA vs. DC/HC, increase	Soybean agglutinin	GalNAc
STL	RA vs. DC, increase	Solanum tuberosum lectin	GlcNAc
SNA	RA vs. DC, increase	Sambucus nigra lectin	Sialic acid
Jacalin	RA vs. DC, increase	Jacalin	Galβ3GalNAc
PHA-E	RA vs. DC, increase	Phaseolus vulgaris Erythroagglutinin	Galβ4GlcNAc
SNA-I	Seropositive vs. Seronegative, increase	Sambucus nigra (Elderberry Bark)	Sialic acid
AAL	Seropositive vs. Seronegative, increase	Aleuria aurantia lectin	Fucose
ConA	Seropositive vs. Seronegative, increase RA-ILD vs. RA-nILD, increase	Con A Lectin	Mannose
MNA-M	Seropositive vs. Seronegative, increase RA-ILD vs. RA-nILD, increase	Morniga M Lectin (black elderberry)	Mannose
LCA	RA-ILD vs. RA-nILD, increase	Lens Culinaris Agglutinin	Fucose
PHA-L	RA-ILD vs. RA-nILD, decrease	Phaseolus vulgaris Leucoagglutinin	Galβ4GlcNAc
DBA	Remission vs. High disease activity, decrease	Dolichos biflorus agglutinin	GalNAc

Lectin microarray results were further explored across different RA subgroups (Table 1), and results were illustrated in Fig. 2: (1) Significantly higher glycan levels of sialic acid (recognized by SNA-I), mannose (recognized by MNA-M and ConA), fucose (recognized by AAL), were observed for seropositive patients compared to the seronegative group (p < 0.05). (2) Significantly higher glycan levels of mannose (recognized by MNA-M and ConA), fucose (recognized by LCA) while lower glycan levels of Galβ4GlcNAc (recognized by PHA-E and PHA-L) were observed for RA-ILD patients compared to the RA-nILD group (p < 0.05). (3) Significantly higher glycan level of GalNAc (recognized by DBA) was observed for patients that categorized as remission (DAS28 ≤ 2.6) compared to the high disease activity (DAS28 > 5.1) group by using both standards of DAS28-ESR and DAS28-CRP (p < 0.05).

### Validation of glycosylation changes of IgG by lectin blot

IgG heavy chains were selected in lectin blot to verify the microarray results. The intensity of the following lectins on serum IgG from related groups were analyzed: (1) STL, PHA-E, SNA, Jacalin in groups of RA patients and DC patients, (2) SNA-I and ConA in subgroups of RA-seropositive and RA-seronegative, (3) LCA and PHA-L in subgroups of RA-ILD and RA-nILD, (4) DBA in subgroups of remission and high disease activity, and no significant results were observed.

**Fig. 3:**
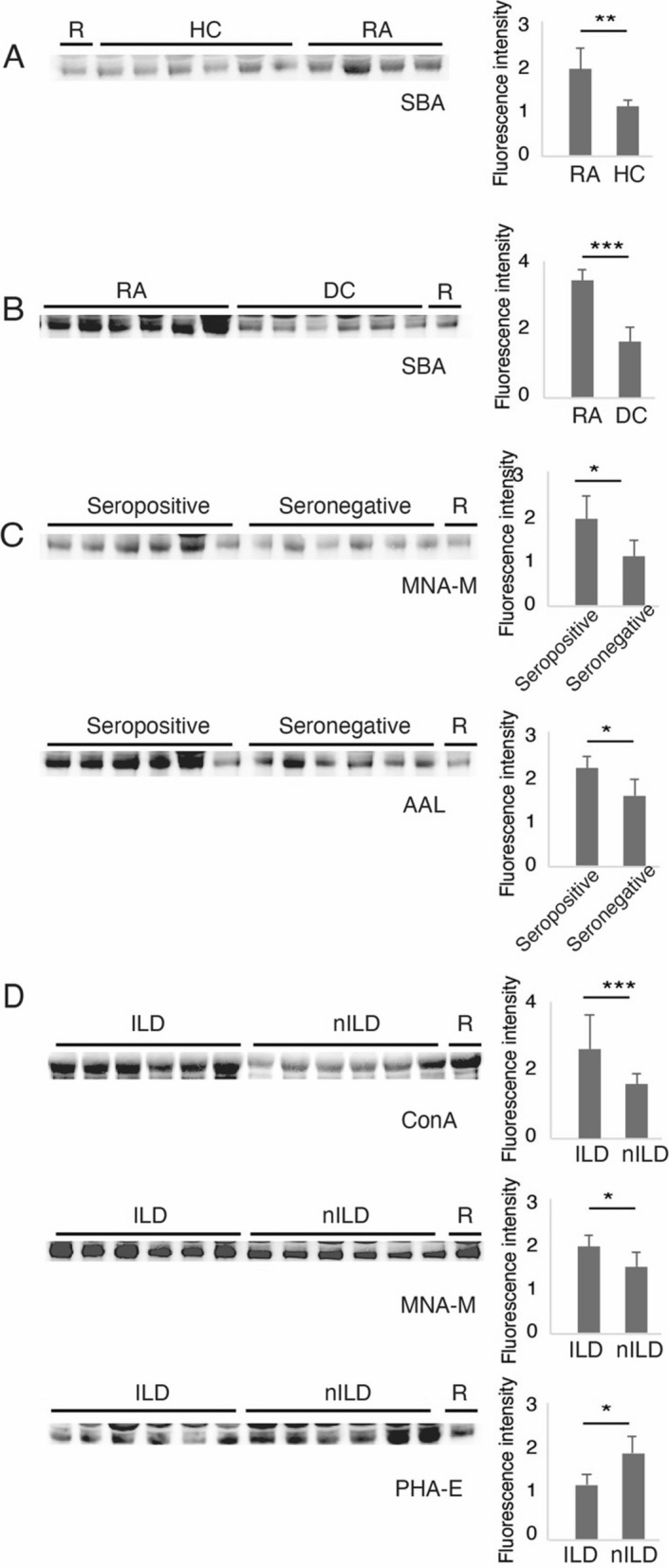
Lectin blot of lectins for serum IgG in RA/DC/HC groups and RA subgroups. Comparison of fluorescence intensity of lectin blot bands are showed in bar graph. R, reference; *p < 0.05, **p < 0.01, ***p < 0.001

For groups of RA versus HC and RA versus DC patients, 24 serum samples were randomly selected for SBA lectin blot validation, and results showed that the intensity of SBA on serum IgG from RA patients was significantly increased compared to either HC or DC patients (p < 0.05) (Fig. 3A and B). For RA subgroups, at least 18 serum samples from each group were chosen for validation, and a new cohort of 50 RA-ILD patients was involved in the selection. The results were listed as follows: (1) MNA-M and AAL lectins were applied to recognize glycans of serum IgG in RA-seropositive and RA-seronegative groups, and increased intensities were observed in RA-seropositive samples (p < 0.05) (Fig. 3C); (2) Lectins of ConA, MNA-M and PHA-E were applied to recognize glycans of serum IgG in RA-ILD and RA-nILD groups, and increased intensities of ConA, MNA-M as well as decreased intensities of PHA-E were observed in RA-ILD samples (p < 0.05) (Fig. 3D). These results were consistent with those from lectin microarrays, which confirmed the reliability of lectin microarray analysis. The consistent summary of verification results was shown in Table 2.

### Candidate biomarkers for the diagnosis of RA and RA-ILD

**Fig. 4:**
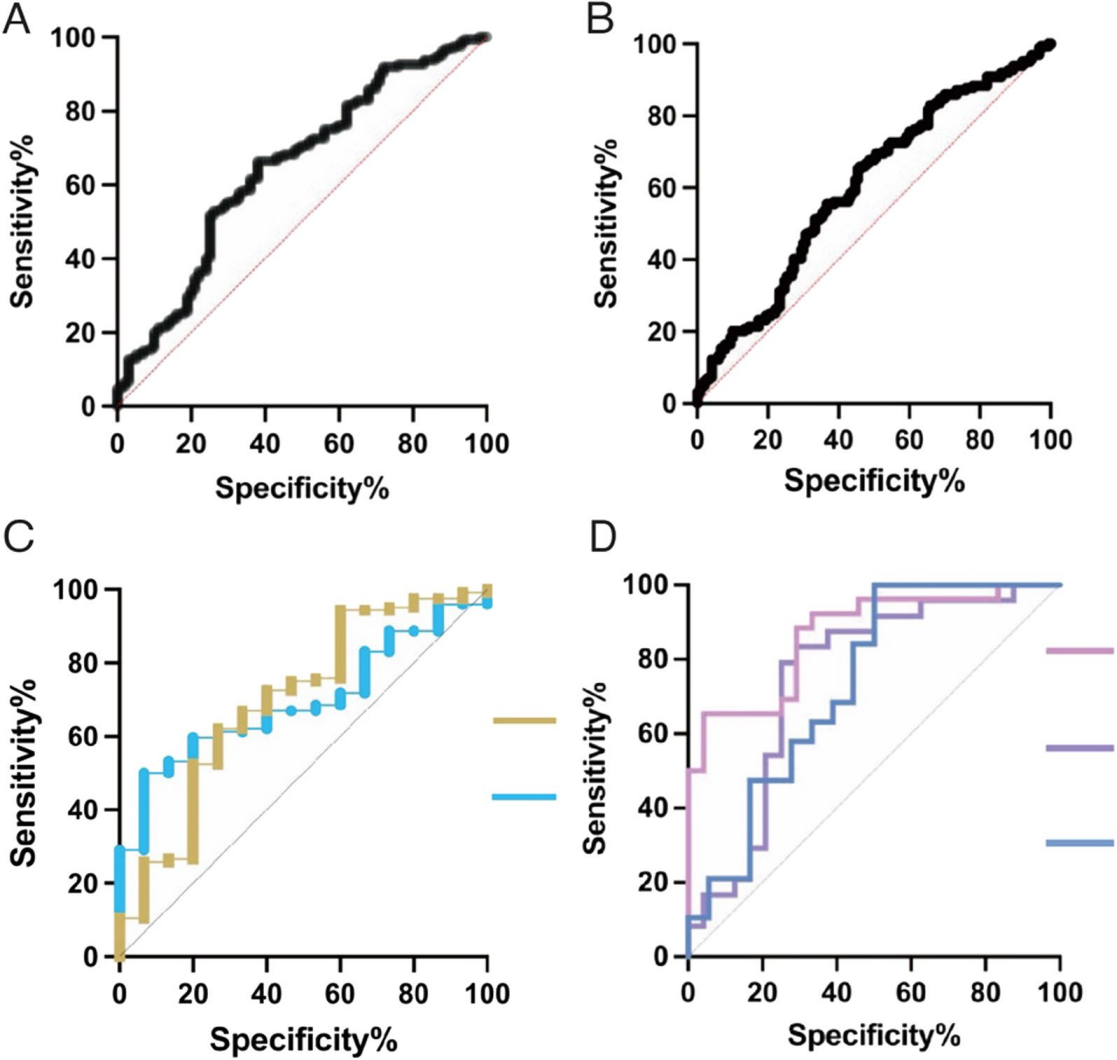
The ROC curve of the biomarkers for the classification of **A** RA/HC **B** RA/DC **C** RA-seropositive and RA-seronegative **D** RA-ILD and RA-nILD

The prediction models of sensitivity and specificity were analyzed as described in the method, and the ROC curves were further constructed for the identified lectin biomarkers. Both data of lectin microarray and lectin blot were applied for the prediction model, and results showed that: (1) Based on the data of lectin microarray in the groups of RA/HC and RA/DC, the diagnosis of RA by lectin SBA showed a sensitivity of 66.46% and a specificity of 62% combined with an AUC of 0.65 (J = 1.362, p < 0.0001, Fig. 4A), and (2) a sensitivity of 65.24% and a specificity of 54.67% combined with an AUC of 0.61 (J = 1.376, p = 0.001, Fig. 4B), respectively. (3) By analyzing the data of lectin blot in the subgroups of seropositive and seronegative, the lectins of AAL (sensitivity = 62.1%, specificity = 73.33%, AUC = 0.70, J = 2.051, p = 0.01) and MNA-M (sensitivity = 50%, specificity = 93.33%, AUC = 0.70, J = 3.21, p = 0.01) could be used as alternative biomarkers for seropositive (Fig. 4C). (4) Data of lectin blot in the subgroups of RA-ILD and RA-nILD were applied for the prediction model, and the lectins of ConA (sensitivity = 65.38%, specificity = 95.83%, AUC = 0.87, J = 1.024, p < 0.0001), MNA-M (sensitivity = 79.17%, specificity = 75%, AUC = 0.75, J = 0.95, p = 0.003), PHA-E (sensitivity = 100%, specificity = 50%, AUC = 0.73, J = 1.096, p = 0.02) could be candidate biomarkers for the diagnosis of ILD in RA patients (Fig. 4D).

## Discussion

RA is a common prevalent chronic inflammatory disease, and ILD accounts for one of the most life-threatening complications for RA patients with high mobility [3]. To date, numerous studies have confirmed the important roles of immunoglobulin glycosylation, especially the Fc fragment of IgG that involved in multiple immune processes [22–25]. Aberrant IgG glycosylation profiles have been observed in kinds of AIDs and proved to be involved in their pathogenesis [26–29]. Especially in the case of RA, the glycosylation disturbances of IgG were strongly associated with changes of disease activity and viewed as a hopeful marker of disease activity [30]. Sialylation levels of IgG in RA contribute to the regulation of arthritogenicity, indicating a potential target of antigen-specific immunotherapy [31]. IgG hypogalactosylation was proved to be more related to RA than that in axial spondyloarthritis, a chronic inflammatory disease without relevant antibody reactivity [31, 32]. And in our study, three kinds of chronic inflammatory diseases without RA antibody reactivity (APS, TA, VD) were included to compare with RA, offering a more comprehensive analysis of differential glycosylation between diseases. In the present study, we performed IgG specific glycosylation detection in a large cohort of RA patients by using a lectin microarray technology containing 56 lectins, which is a newly developed high-throughput, high-speed, and high-specific glycan analysis that widely applied to biomarker identification for diagnosis of tumors as well as AIDs [8, 33–38].

A total of 464 serum samples (214 of RA, 100 of HC, 150 of DC) have been applied to lectin microarray, and 14–28 serum samples of each group were randomly chosen to perform lectin blot for validation. The significant differential glycan binding affinities were calculated by the fluorescence intensity. As a comprehensive analysis of lectin microarray and lectin blotting, our study showed that compare to HC and DC groups, serum IgG from RA patients had a higher affinity to the SBA lectin. For RA subgroups, RA-seropositive group had higher affinities to the lectins of MNA-M and AAL, and RA-ILD group had higher affinities to the lectins of ConA and MNA-M while a lower affinity to the PHA-E lectin. For those results of lectin blotting that were not consistent with the results of lectin microarray, it might be due to the limited number of tested specimens or excessive heterogeneity.

To date, serum positives of CCP and RF have been considered as pivotal biomarkers for the diagnosis of RA [5], while we offered an alternative diagnostic approach of lectin microarray technology, especially for screening of large number of samples. Higher glycan levels of GalNAc (recognized by SBA) were observed in the serum of RA patients compare to HC and DC, and the method of SBA lectin detection was supported by the prediction model of sensitivity and specificity. Furthermore, higher levels of mannose (recognized by MNA-M) and fucose (recognized by AAL) that observed in RA-seropositive patients suggested the underlying mechanisms of glycosylation involved in the production of autoantibodies. In previous studies, the higher fucosylation has been found in serum of RA patients compared with healthy controls, and our results give a further indication that it is even higher in RA-seropositive patients [39, 40].

Considering the serious clinical outcomes of ILD and the urgent need of diagnosis for ILD in RA patients [3], for the first time, our results showed that lectins of ConA, MNA-M, PHA-E could be candidate biomarkers for the diagnosis of RA-ILD. The prediction models showed high sensitivity (ranges from 65.38% to 100%) and specificity (ranges from 50%, to 95.83%), which supported their application in the diagnosis of RA-ILD. Similarly, significant higher glycan levels of mannose (recognized by MNA-M and ConA) and lower glycan levels of Galβ4GlcNAc (recognized by PHA-E) in RA-ILD patients indicated the potential mechanisms of the pathogenesis of ILD in RA patients.

In conclusion, our study made contribution to decipher the underlying pathogenesis related to glycosylation of RA and provided candidate RA as well as RA-ILD biomarkers for clinical application in the future.

## Supplementary Information

**Additional file 1. Diagram of lectin microarray and heat map of lectin results: Fig. S1.** Lectin microarray technology containing 56 lectins to depict the glycosylation profile of RA. The red fluorescence indicates the binding signal of serum IgG and lectin. **Fig. S2.** Heat map of 56 lectin results from microarray analysis. Rows: HC, DC, RA, and RA subgroups; columns: lectins. Preferred binding sugars for lectins were listed for each lectin. Color key indicates standardized fluorescent intensity for lectins, blue: lowest; red: highest. The heatmap was generated using the heatmap package (version 1.0.8) of the R software (version 3.2.2).

## Data Availability

All data generated or analysed during this study are included in this published article.

## Abbreviations

ACPA: Anti–citrullinated protein antibody
AIDs: Autoimmune diseases
APS: Anti-phospholipid syndrome
AUC: Area under curve
CCP: Cyclic citrullinated peptide
DAS: Disease activity score
DC: Disease control
ESR: Erythrocyte sedimentation rate
HC: Healthy control
ILD: Interstitial lung disease
RA: Rheumatoid arthritis
RF: Rheumatoid factor
ROC: Receiver operating characteristic
S/N: Signal-to-noise ratio
TA: Takayasu arteritis
VD: Vascular disease
