# Supplementary material for: Changes of serum IgG glycosylation patterns in rheumatoid arthritis
Source: Clin Proteomics. 2023 Feb 21;20:7. doi: 10.1186/s12014-023-09395-z (PMC9942403; doi:10.1186/s12014-023-09395-z)
Supplement: Supplementary file 1 — Additional file 1. Diagram of lectin microarray and heat map of lectin results: Fig. S1. Lectin microarray technology containing 56 lectins to depict the glycosylation profile of RA. The red fluorescence indicates the binding signal of serum IgG and lectin. Fig. S2. Heat map of 56 lectin results from microarray analysis. Rows: HC, DC, RA, and RA subgroups; columns: lectins. Preferred binding sugars for lectins were listed for each lectin. Color key indicates standardized fluorescent intensity for lectins, blue: lowest; red: highest. The heatmap was generated using the heatmap package (version 1.0.8) of the R software (version 3.2.2). [file 12014_2023_9395_MOESM1_ESM.docx]

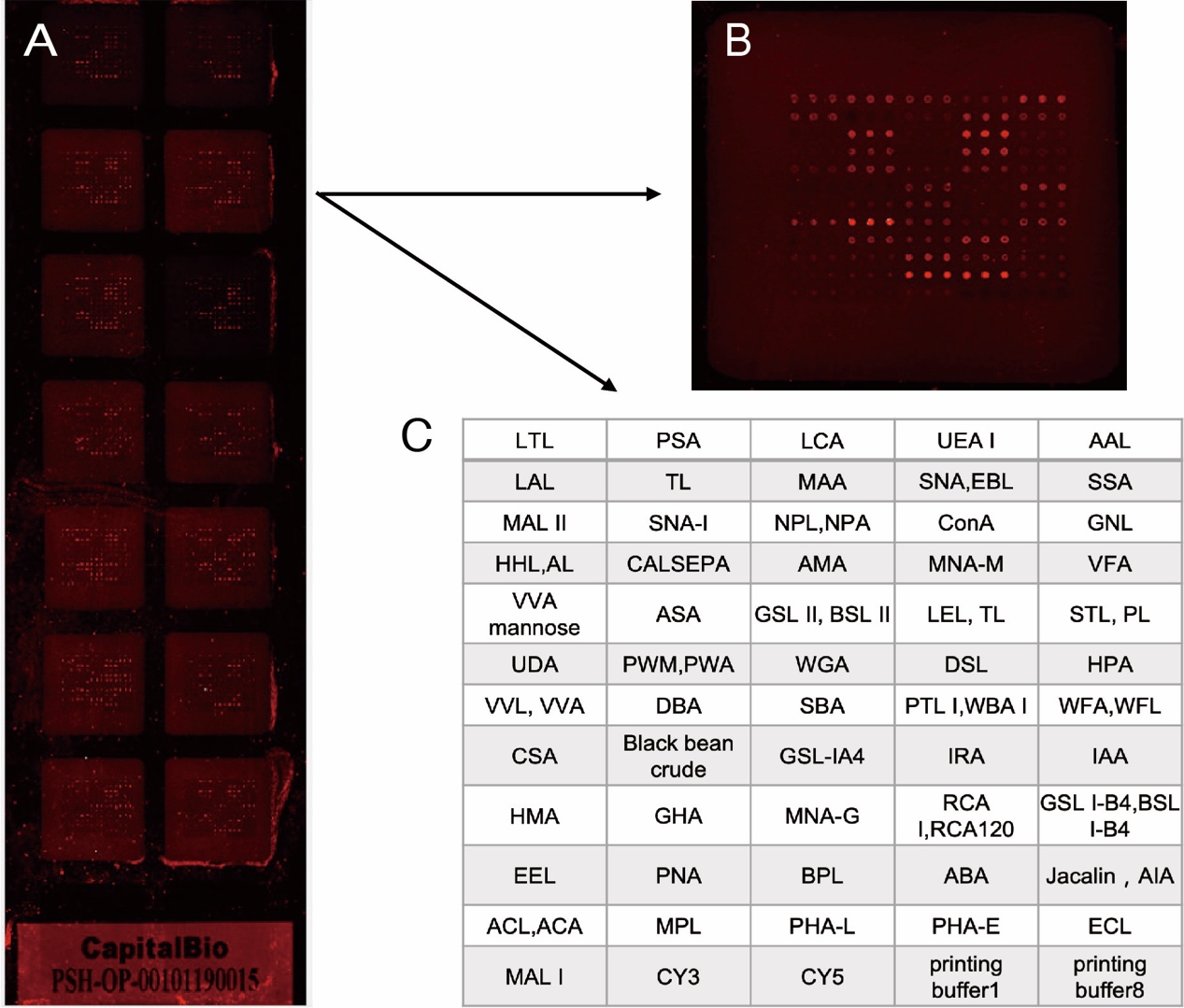


Fig. S1: Lectin microarray technology containing 56 lectins to depict the glycosylation profile of RA. The red fluorescence indicates the binding signal of serum IgG and lectin.


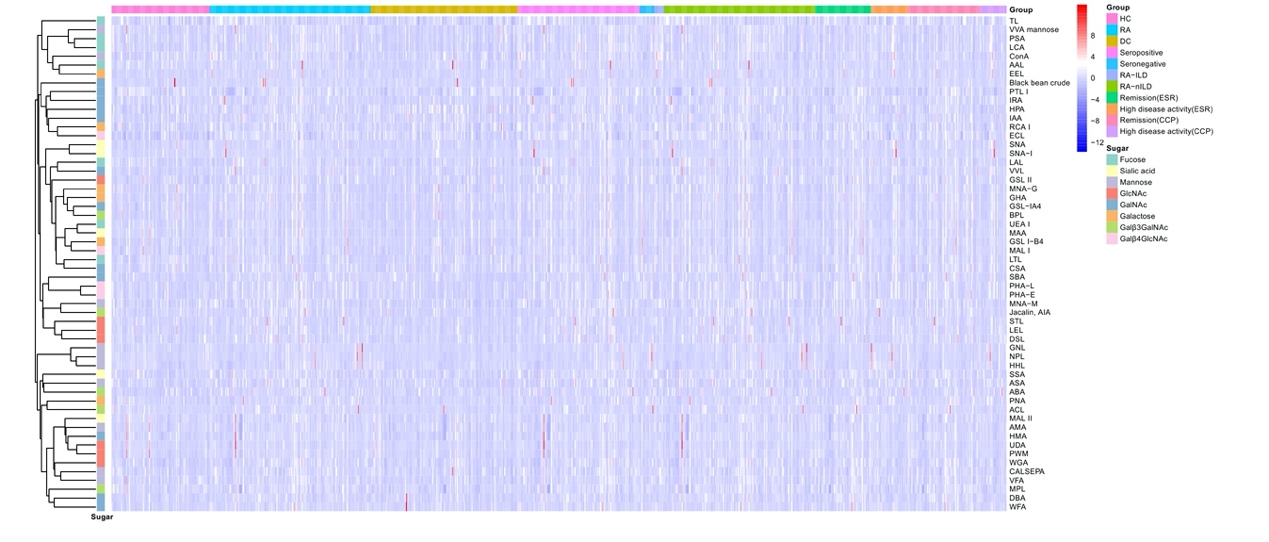


Fig. S2: Heatmap of 56 lectin results from microarray analysis. Rows: HC, DC, RA, and RA subgroups; columns: lectins. Preferred binding sugars for lectins were listed for each lectin. Color key indicates standardized fluorescent intensity for lectins, blue: lowest; red: highest. The heatmap was generated using the heatmap package (version 1.0.8) of the R software (version 3.2.2).
